# Utility of autoimmune serology testing in the assessment of uncharacterized interstitial lung disease: a large retrospective cohort review

**DOI:** 10.1186/s12931-017-0644-4

**Published:** 2017-08-23

**Authors:** Mohammad Alsumrain, Federica De Giacomi, Shireen Mirza, Teng Moua

**Affiliations:** 1 Division of Pulmonary and Critical Care Medicine, Mayo Clinic, 200 First St. SW, Rochester, MN 55905 USA; 2 Dipartimento Cardio-Toraco-Vascolare, University of Milan-Bicocca, Respiratory Unit, San Gerardo Hospital, ASST di Monza; via Pergolesi 33, 20900 Monza, Italy

**Keywords:** Autoimmune serology, Connective-tissue disease interstitial lung disease

## Abstract

**Background:**

Autoimmune serologies are often obtained in the initial evaluation of uncharacterized interstitial lung disease (ILD). Whether this practice is helpful in delineating connective-tissue disease related ILD (CTD-ILD) is not well known. We assessed the frequency of incident CTD-ILD as detected by autoimmune serology testing and presenting clinical signs and symptoms.

**Methods:**

Consecutive patients seen at our institution over a four year period with newly diagnosed uncharacterized ILD and autoimmune serologic testing were included. Serologic assessment was performed as a standardized order set of 13 laboratory tests. Presenting demographics and clinical signs or symptoms suggestive of autoimmune disease were correlated with the presence or absence of positive serology studies and final CTD-ILD diagnoses.

**Results:**

Overall prevalence of newly diagnosed CTD-ILD was 6.9% (42 of 605). Positive serology was seen in 35.2% (213 of 605) of screened ILD. CTD-ILD was diagnosed in 19.2% of those with positive serology, and 52.8% of those with both positive serology and suggestive clinical signs or symptoms. Only 1.4% of those with positive serology and negative review of systems were diagnosed with CTD-ILD. CTD-ILD diagnoses were made more frequently in younger patients ≤60 years with no diagnoses made after the age of 80 (*P* = 0.009). Positive serology in non-CTD-ILD cases did not appear to confer any survival advantage.

**Conclusions:**

The yield of autoimmune serology testing in uncharacterized ILD appears greatest in those with suggestive clinical signs or symptoms on presentation for CTD-ILD.

## Background

The connective tissue diseases (CTDs) are a spectrum of rheumatologic disorders sharing underlying mechanisms of inflammation and immune-mediated organ damage, one of which is interstitial lung disease (ILD) [1–3]. Reported prevalence of CTD related ILD (CTD-ILD) has ranged from 12.4% to 67% at ILD presentation, depending on the series [4–7]. However CTD diagnosis may be subsequent to initial ILD findings, where ILD may be a first manifestation of early or unsuspected CTD. In a report by Mitoo et al. of 114 patients, 15% were diagnosed with subsequent CTD after evaluation for ILD [8]. Kang et al. found 3.8% of patients with positive serology subsequently going on to develop CTD over an average follow-up period of 33 months. There appeared to be no prognostic value with positive serology [9].

Consequently, screening autoimmune serologies are often routinely obtained in initially uncharacterized ILD, with or without clinical suspicion of CTD [10]. The utility of such screening practices (which specific laboratory tests to obtain and in which patients) and its implications for diagnosis and treatment have not been well studied. Nonspecifically positive serologic findings have been reported in idiopathic pulmonary fibrosis (IPF) and may confuse both CTD-ILD and IPF diagnosis [11, 12]. Recent criteria describing interstitial pneumonia with autoimmune features (IPAF) [13] may delineate early or developing CTD-ILD and unclassifiable ILD or IPF, but diagnostic questions remain where clinical signs or symptoms of autoimmune disease present variably, particularly in those with usual interstitial pneumonia-like features (UIP) [14]. Our aim was to study the clinical utility of autoimmune serologies in the detection of undiagnosed CTD-ILD and describe predictive demographic or clinical features, highlighting the frequency of newly diagnosed CTD-ILD in a large cohort of uncharacterized ILD.

## Methods

**Fig. 1:**
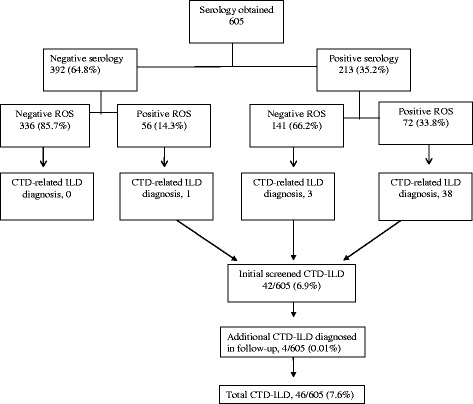
Cohort distribution as stratified by serology and review of systems

Study approval was obtained (Mayo Clinic Institutional Review Board # 16–006286). The records of 2623 consecutive new referrals to our ILD Clinic between 1/1/2009 and 12/31/2012 were reviewed for this study. Patients without autoimmune serology testing and those with established CTD-ILD at the time of referral were excluded. Those referred for sarcoidosis or without evidence of ILD were also excluded leaving 605 initially uncharacterized subjects whom underwent serologic testing (Fig. 1). Final ILD diagnoses were made by expert pulmonologists based on clinical presentation, radiologic findings, and available histopathological studies, often in a multidisciplinary manner but led by an initial consulting clinician. IPF and other idiopathic interstitial pneumonias (IIP) were diagnosed by existing criteria [15, 16]. Final diagnoses of ‘unclassifiable ILD’ were defined as those with clinical and radiologic features on presentation inconsistent with strict IPF criteria but not meeting definitions of other ILD [17]. IPAF was diagnosed by recent consensus statement [13]. Final CTD-ILD diagnoses were made or confirmed by formal Rheumatology consultation during the initial period of ILD assessment based on standardized criteria (specific CTD included rheumatoid arthritis (RA), Sjögren’s syndrome, scleroderma, mixed connective-tissue disease (MCTD), dermatomyositis/polymyositis or anti-synthetase syndrome, and systemic lupus erythematosus (SLE)). For the purposes of our study, undifferentiated connective-tissue disease-ILD (UCTD-ILD) was not included as a separate CTD-ILD diagnosis and combined with IPAF disease incidence. Other categories of diagnosed ILD included non-IPF idiopathic interstitial pneumonia, hypersensitivity pneumonitis, drug-induced, occupational or exposure-related ILD, sarcoid, pulmonary vasculitis, and other rare lung diseases (lymphangioleiomyomatosis, amyloidosis, Burt-Hogg-Dube, etc.).

Autoimmune serologic assessments were defined as studies obtained on initial work-up of uncharacterized ILD with or without positive review of systems (ROS) for autoimmune disease. An institutional order set was previously established by our ILD Clinic practice for CTD screening, consisting of 13 laboratory tests (see Table 1 for institutional ranges and positive cut-offs). Detection of pulmonary vasculitis was also included in this screening cohort, though final vasculitis diagnoses were not considered newly diagnosed CTD-ILD for the purposes of this study. Other autoimmune laboratory studies not included in the screening panel (anti-centromere, extended anti-synthetase antibody panel, etc.) were considered obtained for diagnostic purposes and not included in this study. Given their specific assessment of particular diseases of interest, they were considered more diagnostic than screening..

**Table 1:** Autoimmune Serology Screening Panel

Test Name	Reference Values	Screening Positive Cut-off
ANA, serum	< or =1.0 U (negative), 1.1–2.9 U (weakly positive), 3.0–5.9 U (positive) ≥ 6.0 U (strongly positive)	>1.0 U
SS-A/Ro antibody, serum IgG	<1.0 (negative)	≥1.0 (positive)
SS-B/La antibody, serum IgG	<1.0 (negative)	≥1.0 (positive)
Smith antibody, serum IgG	<1.0 (negative)	≥1.0 (positive)
RNP antibody, serum IgG	<1.0 (negative)	≥1.0 (positive)
Scl 70 antibody, serum	<1.0 (negative)	≥1.0 (positive)
Jo-1 antibody, serum IgG	<1.0 (negative)	≥1.0 (positive)
RF, serum antibodies (all classes against Fc of IgG)	<15 IU/mL	>15 IU/mL
CCP antibody, serum	<20.0 U (negative), 20.0–39.9 U (weak positive), 40.0–59.9 U (positive), ≥ 60.0 U (strong positive)	>20 U
MPO antibody, serum	<0.4 U (negative), 0.4–0.9 U (equivocal), ≥1.0 U (positive)	≥1.0 U
PR3 antibody, serum	<0.4 U (negative), 0.4–0.9 U (equivocal), ≥1.0 U (positive)	≥1.0 U
Creatinine kinase, serum	Male >18 yo: range 52–336 U/L Female >18 yo: range 38–176 U/L	>336 U/L (male) >176 U/L (female)
Aldolase, serum	<7.7 U/L	>7.7 U/L

*Abbreviations*: *ANA* Antinuclear antibody, *CCP* Cyclic citrullinated peptide, *IgG* immunoglobulin G, *MPO* Myeloperoxidase, *PR3* Proteinase 3, *RF* Rheumatoid factor, *RNP* ribonucleotide protein, *Scl 70* scleroderma topoisomerase 70, *SS-A* anti-Sjögren’s syndrome A, *SS-B* anti-Sjögren’s syndrome B

Suggestive presenting signs or symptoms of autoimmune disease or CTD as documented by clinicians included 1) Raynaud’s phenomenon, 2) sicca symptoms (recurrent dry eyes or mouth), 3) arthralgias, synovitis, joint swelling, or stiffness, 4) unexplained painless or non-pruritic rash or photosensivity, 5) myalgias or specific muscle weakness, 6) mechanic’s hands, 7) Gottron papules, 8) dysphagia, and 9) unexplained constitutional symptoms of chronic or recurrent fatigue, malaise, or fever. Patient demographics, smoking history, and pulmonary function (PFT) testing at presentation (percent predicted total lung capacity (TLC %), forced vital capacity (FVC %), and diffusion capacity for carbon monoxide (DLCO %)) were also collated. Frequency of positive and negative serology findings were correlated with suggestive findings on clinical review of systems (ROS) and final ILD diagnoses.

Cohort follow-up was defined from the date of initial clinic visit to date of last follow-up or death, in months (median and interquartile range (IQ range)). Interval incident diagnoses of CTD-ILD were collated and defined similarly for initial screening cases, requiring confirmation by expert Rheumatology consultation.

### Statistical methods

Data were presented as mean and standard deviation (SD) or median and interquartile range (IQR) for continuous variables and as counts and percentages for categorical variables. For comparisons, chi-square or Fisher’s exact test were used for categorical variables and two sample t-test for continuous variables. Kaplan-Meier analysis with Log-rank test was used for comparing survival among selected groups (non-CTD-ILD positive vs negative serology cohorts). Statistical analysis was performed using SPSS Software (Version 20.0, IBM USA), with two-sided *p*-value <0.05 considered statistically significant.

## Results

Autoimmune serologies were obtained in six hundred and five patients with initially uncharacterized ILD with one or more laboratory tests returning positive in 213 (35.2%) (Fig. 1). Positive ROS for signs or symptoms of autoimmune disease were seen in 72 (33.8%) and 56 (14.3%) of those with positive and negative serology, respectively. CTD-ILD was diagnosed in only 19.2% of those with positive serology, but found in 52.8% of those with both positive serology and suggestive clinical signs or symptoms of autoimmune disease. Remaining subjects with positive serology and ROS were diagnosed as IPAF in 7, vasculitis in 4, IPF in 4, other IIP in 6, unclassifiable ILD in 8, hypersensitivity pneumonitis in 2, sarcoid in 1, and rare lung diseases in 2 (non- Sjögren’s related amyloidosis). CTD-ILD was diagnosed in 3 patients (1.4%) with positive serology and negative ROS. One had subtle skin glossiness around his fingers, highly positive Scl-70, NSIP CT pattern, and subsequent capillaroscopy consistent with scleroderma though presented with no initial extra-pulmonary symptoms including Raynaud’s phenomenon. The two remaining patients had NSIP-like or NSIP/organizing pneumonia overlap patterns on CT with serology consistent for Sjögren’s syndrome despite absent extra-pulmonary symptoms. Lip biopsies confirmed disease in both.

Initial clinical diagnoses for the whole cohort are presented in Table 2. IPF was the most frequent diagnosis (32.6%) followed by ‘unclassifiable’ ILD (24.1%), and other idiopathic interstitial pneumonia (IIP) (15.2%). Total CTD-ILD diagnosis was made in 7.6% of the whole cohort (46 of 605) (Fig. 1).Frequency of positive screening serology among non-CTD, non-IPAF, and non-vasculitis patients, ranged from 22 to 40%. Positive clinical signs or symptoms suggestive of autoimmune disease was greatest in those with final CTD-ILD (90%), followed by IPAF (72%) and ANCA-associated vasculitis (80%).

**Table 2:** Final clinical diagnoses and distribution of positive autoimmune serologies and suggestive clinical signs or symptoms of autoimmune disease

ILD type	Serology obtained (*N* = 605)	Positive serology^b^	Positive clinical signs or symptoms suggestive of autoimmune disease^c^	Duration of disease cohort follow-up, months (median (IQR))	Subsequent development of CTD after initial visit, N (%)
IPF (N, (%))	197 (32.6)	51 (25.8)	19 (6.9)	9.2 (1.05–35.1)	0
Unclassifiable ILD (N, (%))	146 (24.1)	46 (31.5)	24 (10.8)	12.6 (0.3–47.5)	3
Other IIP (N, (%))	92 (15.2)	24 (26.1)	21 (15.9)	9.7 (0.6–52.8)	1
Hypersensitivity pneumonitis (N, (%))	70 (11.6)	22 (31.4)	10 (9)	9 (0.6–44.7)	0
CTD-ILD ((N, (%))	42 (6.9)	41(98)	38 (90)	14.8 (0.9–66.6)	-
Drug induced/Environmental (N, (%))	15 (2.5)	4 (26.7)	0	6.2 (1.7–50)	0
Rare ILD^a^ (N, (%))	11 (1.8)	3 (27)	2 (7.1)	16.2 (0.1–59.7)	0
IPAF (N, (%))	18 (2.9)	14 (78)	13 (72)	17.3 (1.8–55.1)	0
Sarcoidosis (N, (%))	5 (0.82)	2 (40)	1 (9)	4.1 (0.6–33.8)	0
CPFE (N, (%))	4 (0.66)	2 (50)	0	28.4 (3.3–55.1)	0
Aspiration-related fibrosis (N, (%))	1 (0.16)	0 (0)	1 (16.6)	55.7 (−)	0
ANCA vasculitis related ILD (N, (%))	4 (0.66)	4 (100)	4 (80)	80.9 (25–96.7)	0

*Abbreviations*: *ANCA* anti-neutrophilic cytoplasmic antibodies, *CTD-ILD* connective tissue disease-related interstitial lung disease, *CPFE* combined pulmonary fibrosis and emphysema, *IIP* idiopathic interstitial pneumonia, *ILD* interstitial lung disease, *IPAF* interstitial pneumonia with autoimmune features, *IPF* idiopathic pulmonary fibrosis, *IQR* interquartile range

^a^Rare ILD includes lymphangioleiomyomatosis, pulmonary Langerhans cell histiocytosis, pulmonary amyloidosis, pulmonary alveolar proteinosis, Williams-Campbell syndrome, IgG4 sclerosing lung disease, ILD in dyskeratosis congenita, Birt-Hogg-Dubé syndrome

^b^percentages are positive laboratory findings over those with obtained serology

^c^percentages are for positive review of systems for the disease type, inclusive of serology screened and non-screene

Median follow-up for the whole cohort was 11 months (IQ range 0.6–46.1) (Table 2). A little under half (*N* = 292 (48%)) had at least 12 months or more of follow-up, while only 225 (37%) had greater than 24 months of follow-up. Incident diagnoses of CTD-ILD after initial clinic visit in the screened cohort occurred in only 4 patients (0.007%) (Table 4). One was initially diagnosed as suspected desquamative interstitial pneumonia (DIP) based on CT and heavy smoking history, though had nonspecific joint symptoms and mildly positive ANA at presentation. He was diagnosed with rheumatoid arthritis 53 months after initial visit. The other three were originally diagnosed as unclassifiable ILD or ILD of unknown etiology. Two of the three had negative ROS and serology at initial visit, while one had positive rheumatoid factor and reported dysphagia. The two with negative serology and ROS were subsequently diagnosed as limited scleroderma and rheumatoid arthritis at 61 and 48 months respectively, with the last subject diagnosed with mixed-connective tissue disease at 12.9 months after initial visit.

Clinical comparisons between diagnosed CTD-ILD (*N* = 42) at screening and serology screened non-CTD patients (*N* = 563) are presented in Table 3. CTD-ILD patients were younger (*P* = 0.001) and nonsmokers (*P* = 0.002). Positive serology was more frequent in CTD-ILD (given the nature of most CTD requiring associated serology for diagnosis), yet was still found in 30.5% of non-CTD patients. There was no statistical difference in gender and baseline PFT findings between the two groups. ANA, RF, SS-A/SS-B, and anti-CCP were the most frequently positive serologies for both cohorts. Individual positive serologies were more frequent in CTD-ILD than non-CTD-ILD except for anti-RNP.

**Table 3:** Baseline demographics and clinical findings -for initial CTD-ILD vs non-CTD-ILD diagnoses in serology screened patients

	Diagnosed CTD-ILD (*N* = 42)	Non-CTD-ILD (*N* = 563)	*P*-value
Age (mean ± SD)	61.0 ± 11.0	67.0 ± 11.5	**0.001**
Sex			
Male, N(%)	23 (54.8)	337 (59.9)	0.760
Female, N(%)	19 (45.2)	226 (40.1)	
Smoking			
Nonsmoker, N(%)	21 (50)	219 (38.9)	**0.002**
Ex-smoker, N(%)	19 (45.2)	334 (59.3)	
Active smoker, N(%)	2 (4.8)	10 (1.8)	
ROS positive, N(%)	39 (92.9)	89 (15.8)	**<0.001**
Positive serology	41(97.6)	172 (30.6)	**<0.001**
TLC% (mean ± SD)	71.5 ± 17.1	72.7 ± 16.7	0.471
FVC%(mean ± SD)	67.5 ± 16.3	69.0 ± 19.0	0.278
DLCO% (mean ± SD)	49.0 ± 17.8	51.8 ± 16.1	0.315
Frequency of selected positive serology tests, N(%)			
ANA	29 (69)	82 (14.6)	**<0.001**
RF	13 (31)	38 (6.7)	**<0.001**
SS-A/SS-B	16 (38.1)	22 (3.9)	**<0.001**
Anti CCP	7 (16.7)	10 (1.8)	**<0.001**
Scl-70	6 (14.3)	4 (0.71)	**<0.001**
Anti Jo	3 (7.1)	1 (0.17)	**<0.001**
RNP	3 (7.1)	21 (3.7)	0.274
Positive clinical signs or symptoms, N (%)			
Raynaud’s phenomenon	16 (38)	15 (2.6)	**<0.0001**
Sicca symptoms	7 (16.6)	24 (4.2)	**0.0004**
Arthralgias/synovitis	19 (45)	44 (7.8)	**<0.0001**
Rash/photosensitivity	4 (9.5)	19 (3.4)	**0.04**
Myalgia/weakness	7 (16.6)	14 (2.5)	**<0.0001**
Mechanic hands	2 (4.8)	1 (0.1)	**<0.0001**
Gottron papules	1 (2.3)	0 (0)	**0.0002**
Dysphagia	5 (11.9)	7 (1.2)	**<0.0001**
Fatigue/malaise/fever	4 (9.5)	8 (1.4)	**0.0003**

*Abbreviations*: *ANA* antinuclear antibody, *CCP* Cyclic citrullinated peptide antibody, *CTD* connective tissue diseases, *DLCO%* percent diffusing capacity for carbon monoxide, *FVC%* percent forced vital capacity, *RF* Rheumatoid factor, *ROS* review of systems, *Scl 70* scleroderma topoisomerase 70 antibody, *SD* standard deviation, *SS-A* anti-Sjögren’s syndrome A antibody, *SS-B* anti-Sjögren’s syndrome B antibody, *TLC%* percent total lung capacity, *RNP* ribonucleotide protein antibody

Positive clinical signs or symptoms suggestive of autoimmune disease are presented in Table 3. Arthralgias or joint symptoms were the most common clinical findings (45% of CTD-ILD), followed by Raynaud’s phenomenon (38%) and myalgia and sicca symptoms (16.6% each respectively).

Final screened and follow-up CTD-ILD diagnoses are presented in Table 4. Systemic sclerosis was the most common screened diagnostic group comprising 17 patients (415%) followed by Sjögren’s syndrome (*N* = 9 (21%)) and rheumatoid arthritis (*N* = 8 (19%)).

**Table 4:** Distribution of Final CTD-ILD Diagnoses

Connective-Tissue Disease Type	Number of patients on initial visit (*N* = 42), N (percentage)	Number of patients in follow-up (non-screened) (*N* = 4), N (percentage)
Systemic sclerosis	17 (40.5)	0
Sjögren’s Disease	9 (21.4)	1(25)
Rheumatoid arthritis	8 (19)	2 (50)
Anti-synthetase syndrome and dermatomyositis/polymyositis	4 (9.5)	0
Systemic lupus erythematosus	2 (4.8)	0
Mixed connective-tissue disease	2 (4.8)	1 (25)

Distribution of combined serology status and ROS for signs and symptoms of autoimmune disease at screening are presented in Table 5. Frequency of final CTD-ILD diagnosis (numbers in parentheses) was greatest in those with both positive serology and positive ROS (*N* = 38 (52.8%)), followed by those with only positive serology (*N* = 3 (2%)). No patient with negative serology and negative ROS was diagnosed with CTD-ILD.

**Table 5:** Distribution of Serology and Clinical Review of Systems and Final CTD-ILD Diagnoses

All patients with serology testing ordered	Positive Serology	Negative Serology
	N (eventual number of final CTD-ILD diagnoses)	N (eventual number of final CTD-ILD diagnoses)
Positive ROS	72 (38)	56 (1)
Negative ROS	141 (3)	336 (0)

*Abbreviations*: *ROS* Review of systems

Frequency of positive serology and CTD-ILD diagnoses as stratified by age groups is presented in Table 6. While frequency of positive serology was no different among the three selected groups (ages ≤60, 61–79, ≥ 80 years; 33.5% vs 36.1% vs 34.4% respectively; *P* = 0.846), CTD-ILD diagnoses were made more frequently in younger patients ≤60 (11.2% of all uncharacterized ILD patients within that age range) with no new diagnoses made in patients over the age of 80 at presentation (*P* = 0.009).

**Table 6:** Frequency of Positive Screening Serology and CTD-ILD Diagnoses stratified by older age group

	Age ≤ 60 years(161 patients)	Age 61–79 years(380 patients)	Age ≥ 80 years(64 patients)	*P* value
Serology positive, N (%)	54 (33.5)	137 (36.1)	22 (34.4)	0.846
CTD-ILD diagnosis, N (%)^a^	18 (11.2)	24 (6.3)	0	0.009

Abbreviations: CTD = connective tissue disease

^a^percentages are number of CTD diagnoses over total number of pts. evaluated in that age group (for example in column 1, 18 CTD diagnoses over 161 patients evaluated for age group ≤60 years) = 11.1%)

**Fig. 2:**
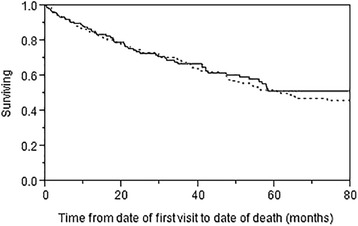
Kaplan Meier Survival Curve Stratified by Positive vs Negative Screening Serology, excluding diagnosed CTD-ILD (*N* = 608)

Finally, Fig. 2 represents Kaplan-Meier assessment of cohort survival as stratified by the presence of positive vs. negative serology findings, after exclusion of initially diagnosed CTD-ILD. There was no difference in survival based on the presence of any positive serology alone (Log-rank 0.63). All-cause mortality for the follow-up period was 27% (*N* = 162) for the whole cohort, greatest among IPF patients (70 deaths, 36%) (Data not shown).

## Discussion

While autoimmune serologies are commonly obtained in the initial work up of uncharacterized ILD, few studies have systematically reviewed the utility of such practices in relation to presenting demographics and clinical findings.

In this large retrospective four-year cohort of uncharacterized ILD, autoimmune serologies were obtained in 605 patients. Total incidence of newly diagnosed CTD-ILD at screening was 6.9%. Of those with positive serology (35.2% of the total cohort), only 19.2% (42 of 213) were ultimately diagnosed with CTD-ILD. Positive serology was seen in 30.6% of non-CTD-ILD cases. Presence of clinical signs or symptoms suggestive of CTD on presentation appeared to increase the yield of laboratory screening, where a little more than half (52.8%) were diagnosed with CTD-ILD by formal rheumatology consultation. With variable short and long-term follow-up (median of 11 months, IQR 0.6–46.1), only 4 additional cases of CTD-ILD were diagnosed (0.007% of the remaining cohort). Overall survival as stratified by the presence or absence of positive serology after exclusion of CTD-ILD cases appeared no different for the remaining ILD subtypes.

It should be emphasized that the yield of autoimmune serologies is likely to be affected by background CTD-ILD incidence and prevalence. In the largest series to date assessing ILD patients seen at a large community hospital between 1999 and 2013, Hu and colleagues found more than two-thirds of their initially uncharacterized ILD patients were eventually diagnosed with either confirmed CTD-ILD or UCTD-ILD [7]. A substantial number were considered misdiagnosed at the time of evaluation. Other centers have reported more modest incidence and prevalence ranging between 12 and 34% of their reviewed cohorts [4–6, 8]. It would stand to reason that the obtaining of autoimmune serologies be tailored to presenting patient demographics where CTD incidence may be expectedly higher or the type of clinical practice (community vs referral) for improving yield and reducing unnecessary testing.

Indeed, a major concern for reflexive or routine autoimmune serology testing is the relatively high frequency of nonspecifically positive results. It is well known that the prevalence of positive laboratory studies such as ANA increase with age, coinciding with the older age of many patients with ILD [18, 19]. Among patients with IPF, the prevalence of nonspecifically positive serology may be as high as 29%, with ANA being the most common [11, 12]. In one retrospective review of IPF patients, a large proportion with positive serology initially received empiric anti-inflammatory or immunosuppressive therapy for suspected CTD-ILD [12], a practice now known to be harmful [20]. Recent criteria for IPAF may also further confound specific CTD-ILD diagnosis, where presenting clinical findings do not meet CTD criteria but suggest early or forme fruste autoimmune disease [13]. We found positive serology in 78% of our patients meeting IPAF criteria, third in frequency only to those with diagnosed ANCA-associated ILD (100% positive ANCA serology testing) and ultimately diagnosed CTD-ILD (98% positive serology). Notably the incidence of diagnosable IPAF in our total cohort (2.9%) was far less than that seen in prior large cohort reviews, owing likely to the retrospective application of current criteria to a historical study period. Recent evidence suggests IPAF patients with UIP-like features on imaging or pathology have similar survival to IPF, further confounding the implications of positive serology among clinically heterogeneous or atypically presenting ILD [14]. With specific treatments available for both IPF and CTD-ILD, distinction of disease is important, often requiring judicious and longitudinal assessment in the interpretation of frequently positive but potentially misleading autoimmune serology studies.

In contrast, negative serology studies may suggest a lower or unlikely probability of current or subsequent CTD-ILD. Our study found no patient with negative ROS and negative serology to have autoimmune disease at presentation, though with variable follow-up, two cases did go on to develop CTD-ILD on average greater than 2 years later. A limited laboratory set targeted at more commonly presenting autoimmune diseases according to age and gender may provide better initial yield. We also found that the presence of suggestive signs or symptoms improved the yield of positive serology screening. In our study, joint symptoms followed by Raynaud’s and myalgias were the more frequent solicited findings. A standardized patient intake or review of systems highlighting such signs or symptoms may be a better impetus for obtaining screening serology than reflexive screening in their absence. This would be more in line with clinical practice where serology findings are often supportive or adjunct to CTD assessment, requiring additional related clinical findings before a confident diagnosis can be made and before treatment is pursued.

Limited by a retrospective design with variable range of follow-up, we found very few subsequent CTD-ILD diagnoses, on average occurring 43.7 months (range of 12.9 to 61 months) after initial screening visit. A more careful and standardized prospective assessment is needed to determine the frequency and timing of future CTD-ILD among a large cohort of initially uncharacterized ILD, and delineate possible clinical predictors. Survival for positive serology findings alone in non-CTD ILD cases did not differ from those with negative serology, consistent with previous findings from Kang et al. [9].

Several limitations should be noted. First, institutional and even individual practices vary in their selection of screening laboratory studies, of which there is no commonly agreed upon panel or set to unify practices. Our institution’s consensus-based order set allowed for the study of commonly ordered labs, but such individual tests may not be available or applicable to other institutions whose screening panels may be derived of other studies and their combinations. For example, our laboratory panel did not include send out studies such as a myositis panel or other extended testing as suggested or included in the recent IPAF serology domain criteria. Our institutional consensus at the time of panel derivation was that such additional testing was considered more directed or confirmatory rather than screening. Individual tests would need to be reviewed by institutions and practices based on local costs, feasibility, and timeliness of results. Indeed, the costs of broad or reflexive testing in addition to the unnecessary workup or treatment of falsely positive results may be considerations for institutions or groups whose practices may be more resource-limited. Finally, all single-center retrospective studies will have inherent limitations including incomplete data and the inability to associate cause and effect.

## Conclusions

Autoimmune serologies are often obtained in the initial assessment of uncharacterized ILD. We found a relatively high frequency of nonspecifically positive serology with lower incidence of new CTD-ILD. Yield of positive serology was improved by younger age at presentation and positive signs or symptoms suggestive of autoimmune disease. Presence of positive serology alone did not appear to confer any survival advantage in this cohort of heterogeneous disease subtypes.

## Abbreviations

ANA: Antinuclear antibody
ANCA: Anti-neutrophilic cytoplasmic antibodies
CCP: Cyclic citrullinated peptide antibody
CTD: Connective-tissue disease
CT-ILD: Connective tissue disease-related interstitial lung disease
IIP: Idiopathic interstitial pneumonia
ILD: Interstitial lung disease
IPAF: Interstitial pneumonia with autoimmune features
IPF: Idiopathic pulmonary fibrosis
RA: Rheumatoid arthritis
RF: Rheumatoid factor
RNP: Ribonucleoprotein
ROS: Review of systems
Scl-70: Scleroderma70 antibody
SLE: Systemic lupus erythematosus
SS-A: Sjögren’s syndrome-A antibody (Ro)
SS-B: Sjögren’s Syndrome-B antibody (La)
SSc: Systemic sclerosis
UCTD-ILD: Undifferentiated connective-tissue disease interstitial lung disease
